# Physicochemical Evaluation of L-Ascorbic Acid and *Aloe vera*-Containing Polymer Materials Designed as Dressings for Diabetic Foot Ulcers

**DOI:** 10.3390/ma15186404

**Published:** 2022-09-15

**Authors:** Magdalena Kędzierska, Mateusz Jamroży, Sonia Kudłacik-Kramarczyk, Anna Drabczyk, Magdalena Bańkosz, Piotr Potemski, Bożena Tyliszczak

**Affiliations:** 1 Department of Chemotherapy, Medical University of Lodz, Copernicus Memorial Hospital of Lodz, 93-513 Lodz, Poland; 2 Faculty of Materials Engineering and Physics, Cracow University of Technology, 37 Jana Pawła II Av., 31-864 Krakow, Poland; 3 Department of Materials Engineering, Faculty of Materials Engineering and Physics, Cracow University of Technology, 37 Jana Pawła II Av., 31-864 Krakow, Poland

**Keywords:** hydrogels, crosslinking agent, crosslinking density, dressings, L-ascorbic acid, *Aloe vera*, swelling ability, tensile strength, elongation, surface roughness

## Abstract

Hydrogels belong to the group of polymers that are more and more often considered as innovative dressing materials. It is important to develop materials showing the most advantageous properties from the application viewpoint wherein in the case of hydrogels, the type and the amount of the crosslinking agent strongly affect their properties. In this work, PVP-based hydrogels containing *Aloe vera* juice and L-ascorbic acid were obtained via UV-induced polymerization. Next, their surface morphology (via both optical, digital and scanning electron microscope), sorption capacity, tensile strength, and elongation were characterized. Their structure was analyzed via FT-IR spectroscopy wherein their impact on the simulated body liquids was verified via regular pH and temperature measurements of these liquids during hydrogels’ incubation. It was demonstrated that as the amount of the crosslinker increased, the polymer structure was more wrinkled. Next, hydrogels showed relatively smooth and only slightly rough surface, which was probably due to the fact that the modifiers filled also the outer pores of the materials. Hydrogels demonstrated buffering properties in all incubation media, wherein during the incubation the release of *Aloe vera* juice probably took place as evidenced by the decrease in the pH of the incubation media and the disappearance of the absorption band deriving from the polysaccharides included in the composition of this additive. Next, it was proved that as the amount of the crosslinker increased, hydrogels’ crosslinking density increased and thus their swelling ratio decreased. Hydrogels obtained using a crosslinking agent with higher average molecular weight showed higher swelling ability than the materials synthesized using crosslinker with lower average molecular weight. Moreover, as the amount of the crosslinking agent increased, the tensile strength of hydrogels as well as their percentage elongation also increased.

## 1. Introduction

Diabetes belongs to the group of chronic diseases, the incidence of which is constantly increasing year by year. It is estimated that in 2035 the number of patients suffering from this disease will amount to over 252 million [1,2]. The diabetes is related to the numerous side effects and complications wherein one of them is a diabetic foot ulcer (DFU) [3]. This is a very serious problem that, if inappropriately treated, may lead even to the amputation of the affected foot [4]. One of methods of the treatments for this complication or the relief to its symptoms is the application of the adequate dressing materials. Importantly, in recent years a growing popularity in the application of hydrogel dressings may be observed [5,6]. This results from their unique characteristics including biocompatibility, wound exudate sorption capability, as well as the ability to accelerate wound healing processes [7,8,9,10].

There are many studies on developing of hydrogel dressings supporting the diabetic foot healing. For example, Yang et al. developed bioactive hydrogel band-aids based on polyurethane (reinforced additionally with imidazolidinyl urea) and tannic acid. Here, it was demonstrated that such materials showed adequate mechanical properties (including high percentage elongation and break strength), adhesiveness, as well as antioxidant and antibacterial properties, indicating at the same time the great application potential for use as dressings [11]. Next, Zhang et al. proposed hydrogel materials obtained as a result of the reaction of carboxymethyl chitosan with hyaluronic acid and incorporated additionally with Au-Pt alloy nanoparticles. Developed materials showed antibacterial and self-healing properties. Moreover, it was proved that such designed materials stimulated angiogenesis and promoted diabetic wound healing [12]. In turn, Li et al. performed studies on pH-responsive hydrogels containing insulin. Based on the research, it was concluded that developed hydrogels showed biocompatibility and bioactivity, accelerated diabetic foot healing, and were able to release insulin to the wound [13]. Innovative dressings designed as materials promoting diabetic foot ulcers treatment were also investigated by Latańska et al. [14], Liu et al. [15], Chen et al. [16], Wang et al. [17], and Zhu et al. [18], and described in [19,20,21].

The hydrogel matrices may be incorporated with various substances promoting wound-healing processes, thus obtaining the hydrogel dressings with enhanced therapeutical properties [22,23,24,25]. For example, L-ascorbic acid (vitamin C) is considered as a modifier that may improve the potential of hydrogel dressings for diabetic foot treatment. It was demonstrated that this substance accelerates this diabetes’ complication healing, reduces the risk of the amputation of affected limb, and plays a significant role in angiogenesis and synthesis of collagen [26,27,28]. Similar potential as a modifier of hydrogel dressings developed for diabetic foot treatment is shown by *Aloe vera* [29,30]. Thus the development of hydrogel dressings incorporated both with L-ascorbic acid and *Aloe vera* juice may lead to designing dressing materials with high application potential for supporting diabetic foot treatment. This is a reason why the main purpose of the research presented in this article was to develop polyvinylpyrrolidone (PVP)-based hydrogel polymers modified with both previously mentioned additives. These materials were next characterized in detail and the results of performed experiments were discussed wherein the main attention was paid to determining the impact of the average molecular weight of the crosslinking agent used during the photopolymerization process on the properties of hydrogels. The studies performed included determining the hydrogels’ swelling ratios in various simulated physiological liquids, checking the impact of these liquids on hydrogels during their incubation in such environments in temperature-simulating conditions occurring in the human body, as well as verifying the presence of functional groups characteristic for hydrogels’ components via FT-IR spectroscopy. Furthermore, the surface morphology of hydrogels was fully evaluated using both the optical, digital, and scanning electron microscope. Importantly, it was also essential to characterize mechanical properties of hydrogels including their tensile strength and the percentage elongation.

## 2. Materials and Methods

### 2.1. Materials

Polyvinylpyrrolidone (PVP, average molecular weight 10,000 g/mol), L-ascorbic acid (vitamin C, ≥99%, ACS reagent), diacrylate poly(ethylene glycol) (PEGDA 575—average molecular weight 575 g/mol and PEGDA 700—average molecular weight 700 g/mol), and 2-hydroxy-2-methylpropiophenone (97%) were bought in Sigma Aldrich (Saint Louis, MO, USA). In turn, *Aloe vera* juice (100%) was purchased from Herbal Pharmaceuticals (Krakow, Poland). All reagents were applied as received without further purification.

### 2.2. Synthesis of Hydrogel Dressings under the Influence of UV Radiation

As a method of preparation of hydrogels, the photopolymerization process employing the UV radiation was selected. This was due to the short reaction time, low energy demand, no by-products formation, and the possibility of simultaneous sterilization of formed materials via UV radiation, which is particularly important considering their use for biomedical purposes. As a main component of hydrogel matrix, polyvinylpyrrolidone (PVP) was selected wherein *Aloe vera* juice and L-ascorbic acid were used as modifiers. Next, diacrylate poly(ethylene glycol) with an average molecular weight 575 g/mol or 700 g/mol was applied as a crosslinking agent, and 2-hydroxy-2-methylpropiophenone was used as a photoinitiator. In order to obtain hydrogels, the adequate amounts of all reagents were mixed, such prepared solutions were poured down to the reaction vessels (Petri dishes with a diameter of 10 cm) and treated with UV radiation. The photopolymerization was performed for 120 s using EMITA VP-60 (Famed, Lodz, Poland) lamp as a radiation source. Detailed compositions of the hydrogels are presented below in Table 1.

Next, selected physicochemical properties of hydrogels were verified and discussed with a primary focus on the influence of the average molecular weight of crosslinker used during the hydrogels’ synthesis on their physicochemical properties.

### 2.3. The Hydrogels’ Surface Morphology Evaluation via Microscopic Techniques

In order to characterize the surface morphology of hydrogels, microscopic techniques were employed. Firstly, a Delta Optical Genetic Bino (Delta Optical, Warszawa, Poland) optical microscope was applied to take a general look at the materials’ surface.

In the next step, the 4K high-precision VKX-7000 Keyence (Keyence, Osaka, Japan) digital microscope recording high-resolution images was used. The apparatus is equipped with CEO REMAX optical engine and 4K CMOS image sensor, providing the high accuracy and magnification. The analysis was performed both to characterize the surface morphology of hydrogels and to define their roughness profiles with selected roughness parameters.

Finally, hydrogels were investigated using the Jeol 5510LV scanning electron microscope (Jeol Ltd., Tokyo, Japan). Before the study, tested samples were dried for 24 h at 37 °C and sputtered with gold. All microscopic analyses were conducted using round samples with a diameter of 1 cm and at room temperature.

### 2.4. Characterization of Hydrogels in Environments Simulating Conditions Occuring in Human Body

As part of these investigations, hydrogels (round samples with a diameter of 1 cm and weighing approximately 1.0 g) were placed for 12 days in 50 mL of selected physiological liquids. The incubation was performed in simulated body fluid (SBF, isotonic to human blood plasma [31]), Ringer liquid (balanced isotonic crystalloid liquid containing physiological concentrations of the following ions: Ca^2+^, K^+^, Na^+^ and Cl^−^ [32]), 2% hemoglobin solution, and distilled water (as a reference liquid) at 37 °C, simulating the temperature conditions occurring in the human organism. During the incubation period, the pH and temperature of incubation liquids were measured using a multifunctional ELMETRON CX-701 (Elmetron, Zabrze, Poland) meter.

### 2.5. Analysis of the Impact of Simulated Physiological Liquids on the Chemical Structure of Hydrogels Using Fourier Transform Infrared (FT-IR) Spectroscopy

FT-IR spectroscopy was employed to verify the impact of hydrogels’ incubation in simulated physiological liquids on their structure. This technique allows to determine the presence of the functional groups characteristic for tested materials, thus the analysis was performed for round samples with a diameter of 1 cm before and after the incubation to compare the absorption bands occurring on obtained FT-IR spectra. The study was carried out at room temperature using the FT-IR spectrophotometer: Thermo Scientific Nicolet iS5 (manufactured by the Thermo Fisher Scientific company, Waltham, MA, USA). The FT-IR spectra were recorded in the wavenumber range: 4000–500 cm^−1^ (32 running scans at the resolution 4.0 cm^−1^).

### 2.6. Sorption Capacity of Hydrogels

Swelling capability of dressing materials allows them to absorb the wound exudate. This, in turn, may speed up the healing process. Therefore, developed materials were also investigated in terms of this property. The hydrogels’ sorption was verified using the same liquids as in the case of incubation studies, i.e., SBF, Ringer liquid, 2% hemoglobin solution (lyophilized powder of bovine hemoglobin was dissolved in distilled water), and distilled water. Hydrogel round samples with a diameter of 1 cm weighing approximately 1.0 g were dried for 24 h at 37 °C and introduced into the 50 mL of the mentioned liquids. After 1 h, swollen samples were separated from the liquid, weighed again, and introduced again into the absorbed medium. The procedure was repeated after 24 h and 48 h.

The sorption capacity of hydrogels was determined using the swelling ratio (α) calculated using the following Equation (1):

(1) $$\alpha =\frac{\left(m_{p}-m_{s}\right)}{m_{p}}$$

where: *α*—swelling ratio, g/g; $m_{s}$—mass of swollen hydrogel sample, g; $m_{p}$—initial mass of hydrogel sample (before swelling), g.

The study was conducted to provide an answer whether the hydrogels show the swelling capability and, if yes, whether this property depends on the absorbed medium or the sample’s composition.

### 2.7. Studies on the Tensile Strength and the Percentage Elongation of Hydrogels

The mechanical properties of hydrogels including their tensile strength and the percentage elongation were also characterized. The paddle-shaped hydrogels were prepared using a ZCP020 manual blanking press and subsequently dried under pressure (to maintain adequate shape) at 37 °C for 24 h. Next, hydrogel samples were subjected to the analysis performed by the universal testing machine (Shimadzu, Kyoto, Japan). The research included placing the samples between the jaws of the mentioned machine, and moving the jaws of the machine apart from each other until the sample breaks. This allowed to determine the hydrogels’ tensile strength ($R_{m}$) and their percentage elongation (A), which were calculated by means of the following Equations (2) and (3):

(2) $$R_{m}=\frac{F_{m}}{S_{0}}$$

(3) $$A=\frac{100\times \left(I_{u}-I_{0}\right)}{I_{0}}$$

where: $R_{m}$—tensile strength, $F_{m}$—the maximum strength, $S_{0}$—the cross-sectional area of analyzed sample before the analysis, A—the percentage elongation, $I_{u}$—the measuring length after the sample was ruptured, and $I_{0}$—the measuring length of the sample before the measurement.

## 3. Results and Discussion

### 3.1. Results of Hydrogels’ Surface Morphology Analysis via Microscopic Techniques

**Figure 1:**
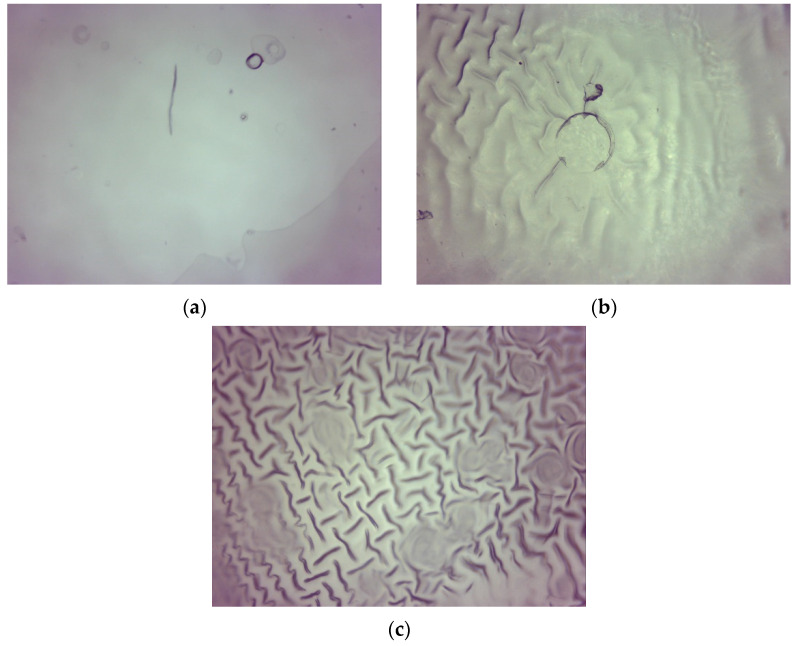
Optical image of sample 575/7.5 (**a**), 575/10.0 (**b**), and 575/12.5 (**c**).

**Figure 2:**
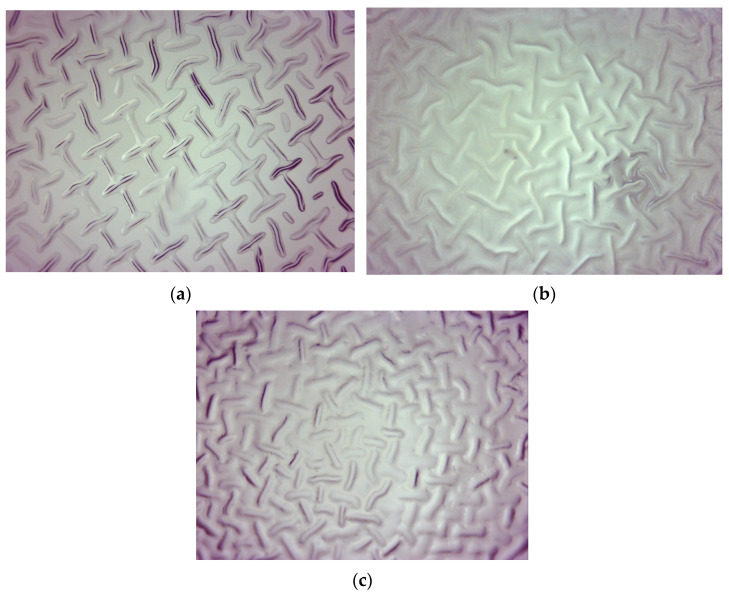
Optical image of sample 700/7.5 (**a**), 700/10.0 (**b**), and 700/12.5 (**c**).

Firstly, the morphology of hydrogels was characterized using the optical microscope. Obtained images are presented below in Figure 1 and Figure 2.

Observations using the optical microscope showed how the amount of the crosslinking agent used during the hydrogels’ synthesis affected their surface morphology. Based on the optical images, it may be noticed that as the amount of the crosslinker in the polymer matrix increased, the concentration of minor irregularities resulting from the polymers’ drying also increased. Importantly, clear differences between the surface morphology of samples 575/12.5 and 700/7.5 may be reported. Next, the observed irregularities on the surface of hydrogels obtained using a crosslinking agent with higher average molecular weight have larger dimensions than these ones observed on the surface of hydrogels prepared using PEGDA 575. It may also be observed that as the amount of the crosslinker in the polymer material increased, the structure of such a polymer became more wrinkled.

**Figure 3:**
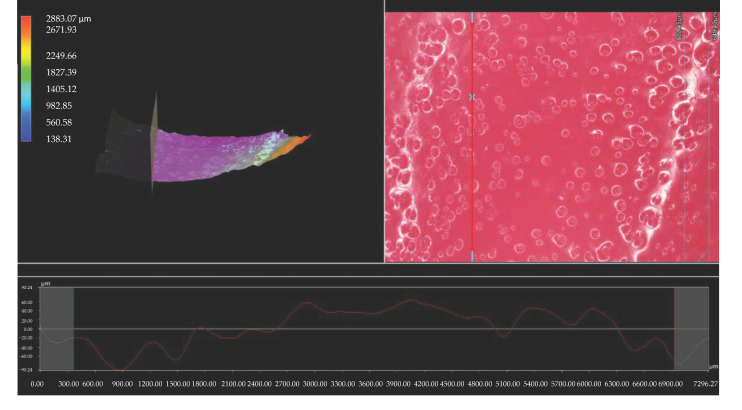
3D view and the roughness profile (along the red line) of sample 575/7.5.

**Figure 4:**
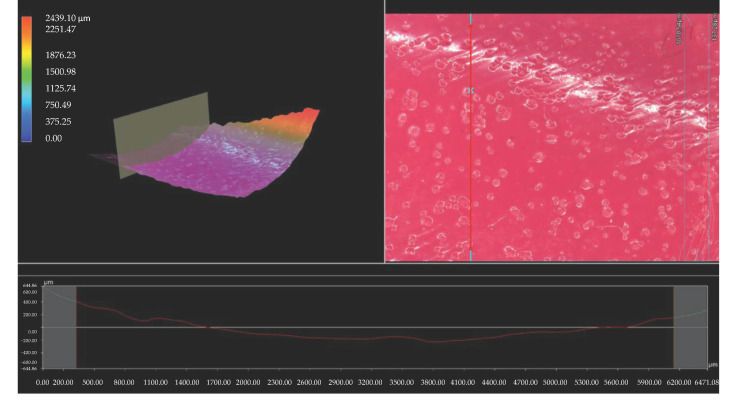
3D view and the roughness profile (along the red line) of sample 575/10.0.

**Figure 5:**
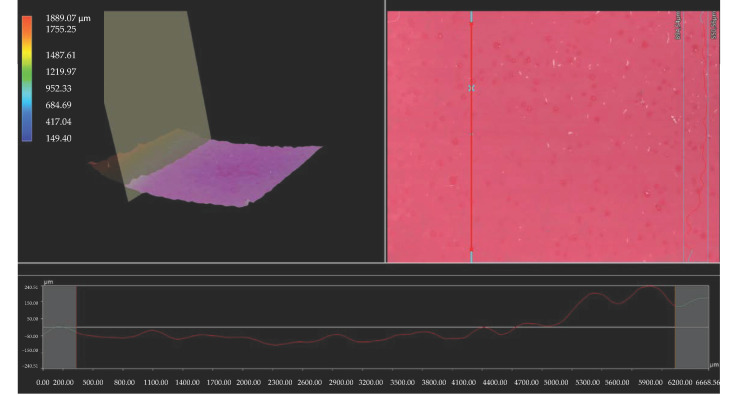
3D view and the roughness profile (along the red line) of sample 575/12.5.

**Figure 6:**
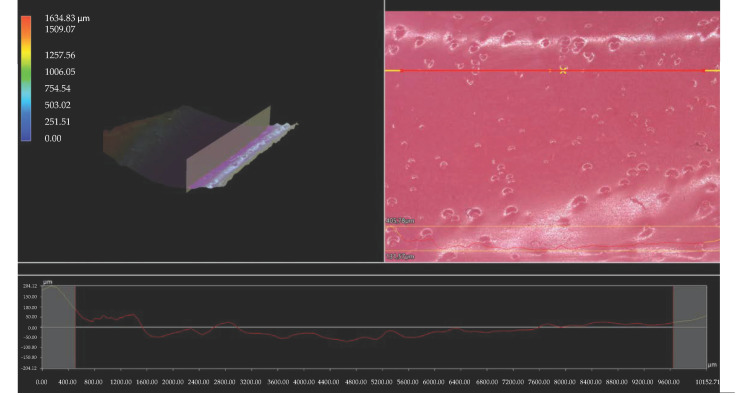
3D view and the roughness profile (along the red line) of sample 700/7.5.

**Figure 7:**
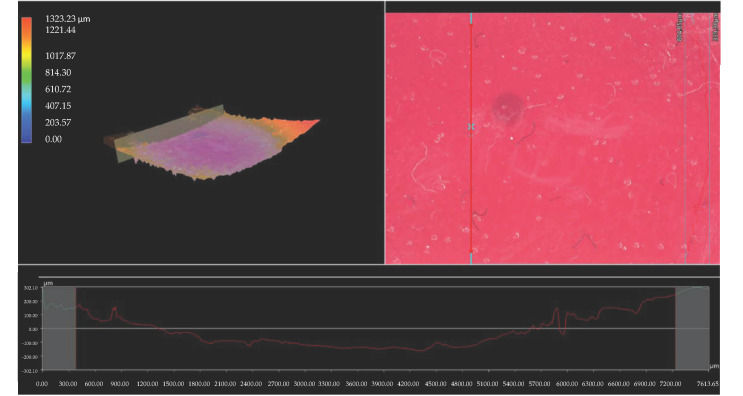
3D view and the roughness profile (along the red line) of sample 700/10.0.

**Figure 8:**
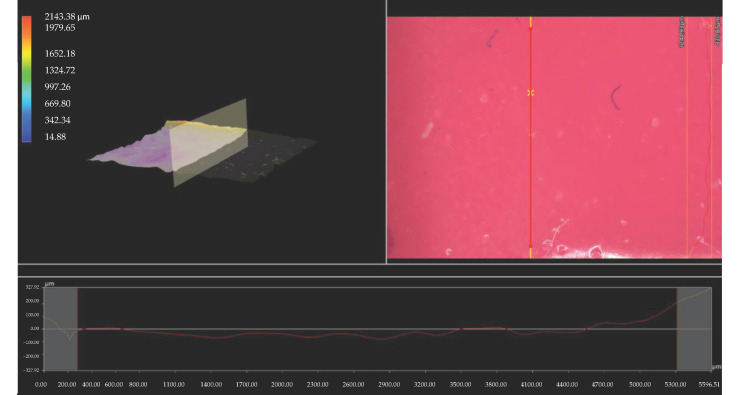
3D view and the roughness profile (along the red line) of sample 700/12.5.

Importantly, the analysis via the optical microscopy was aimed only at generally observing the differences in the hydrogels’ surface morphology. In the next step of the research, a digital microscope was applied to characterize the hydrogels’ morphology as well as their roughness in more detail. The images obtained are presented below in Figure 3, Figure 4, Figure 5, Figure 6, Figure 7 and Figure 8.

We performed an analysis of the materials with various contents of the crosslinking agent with an average molecular weight of 575 g/mol and 700 g/mol allowed for the precise observation of the hydrogels’ surface morphology. Thus, it may be concluded that all tested samples showed relatively smooth and only slightly rough surface. This is probably due to the modification of the hydrogel matrix with *Aloe vera* juice and vitamin C, which filled also the outer pores of the materials. The determined roughness profiles indicate the smooth surface of developed polymers without any sharp irregularities or microcracks. This, in turn, is a beneficial feature of hydrogels in terms of their potential application as dressing materials, as it reduces the risk of their sticking to the patient skin, thereby increasing the comfort of changing of such a dressing [33,34].

What is more, relatively big and visible pores may be observed on the analyzed hydrogel surfaces. Nonetheless, they do not differ significantly from each other despite the different amount of PEGDA with different average molecular weight. They were formed probably by the release of air bubbles from the reaction mixtures during the photopolymerization process and do not result directly from the structure of tested materials.

**Figure 9:**
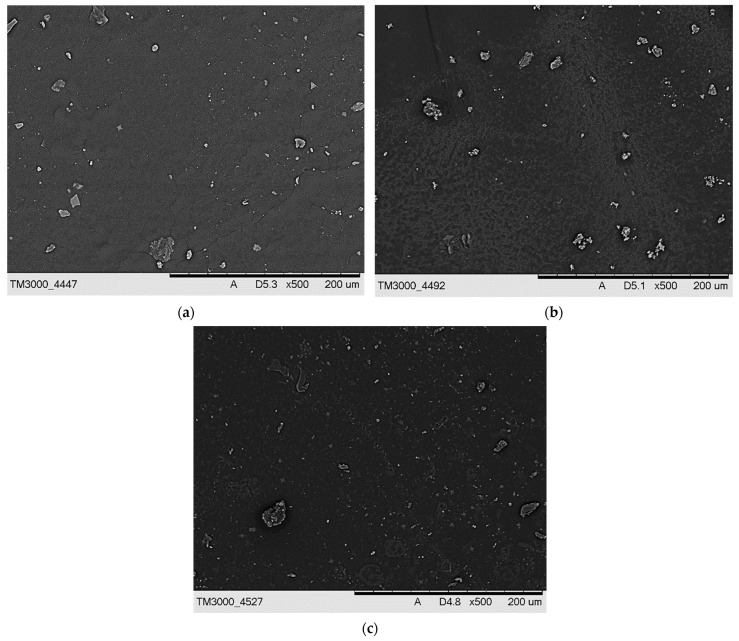
SEM image of sample 575/7.5 (**a**), 575/10.0 (**b**), and 575/12.5 (**c**).

**Figure 10:**
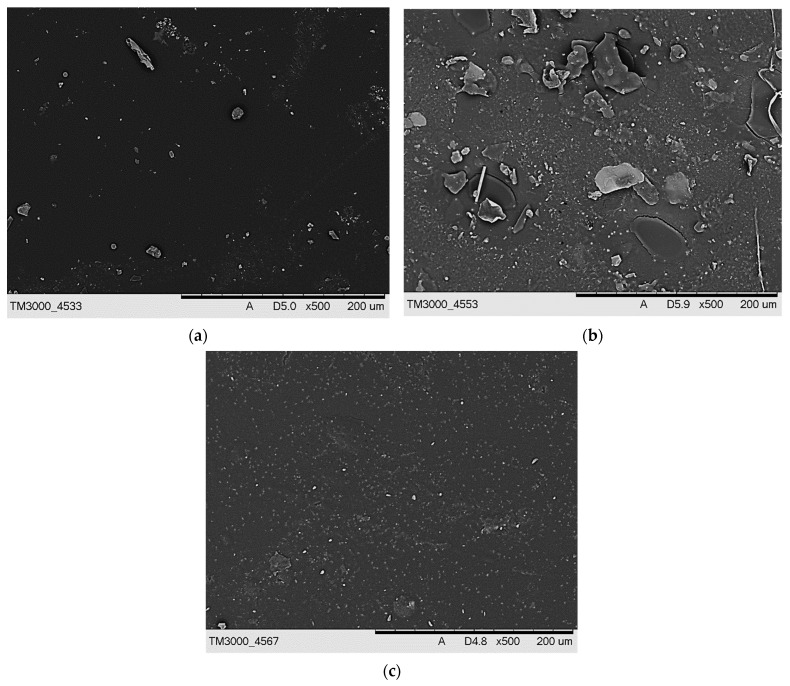
SEM image of sample 700/7.5 (**a**), 700/10.0 (**b**), and 700/12.5 (**c**).

The next analysis included characterizing the hydrogels via SEM technique. The obtained SEM images are presented in Figure 9 and Figure 10.

On the SEM images, any significant differences between the surface morphology of tested hydrogels were not observed. Additionally, any corrugation of the hydrogels’ surface that might be expected as a result of the use of various amount of the crosslinker was also not found. This is probably due to the modification of the hydrogel matrix with *Aloe vera* juice and vitamin C. Probably, the surface morphology analysis of hydrogels after the incubation studies would allow to observe larger differences in the microstructure of the material. Nonetheless, it should be noticed that the results of SEM analysis are consistent with the results of hydrogels’ imaging via the KEYENCE digital microscope.

### 3.2. Effects of Hydrogels’ Incubation in Simulated Physiological Liquids

**Figure 11:**
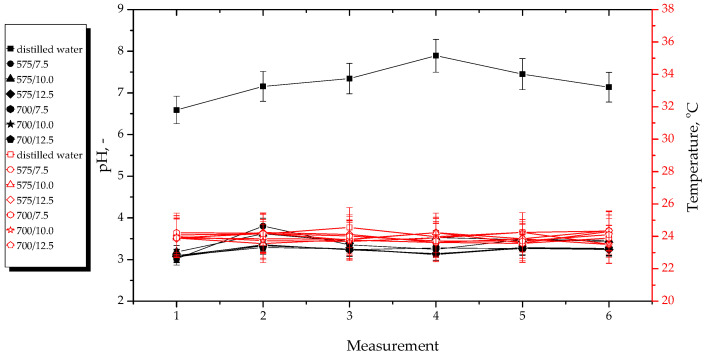
pH and temperature measurements during incubation of hydrogels in distilled water.

**Figure 12:**
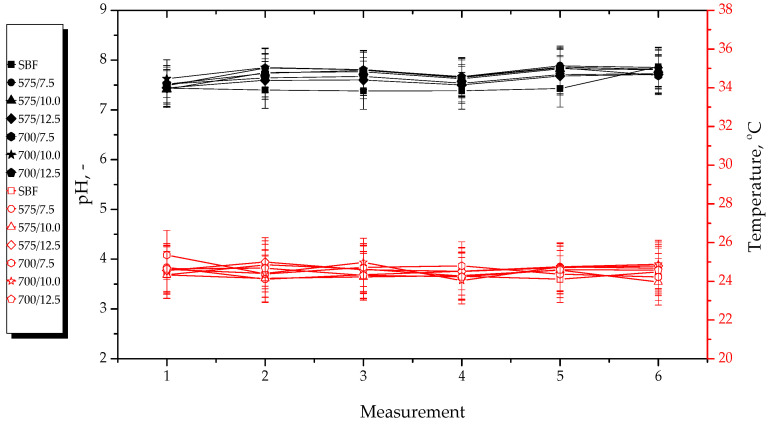
pH and temperature measurements during incubation of hydrogels in SBF.

**Figure 13:**
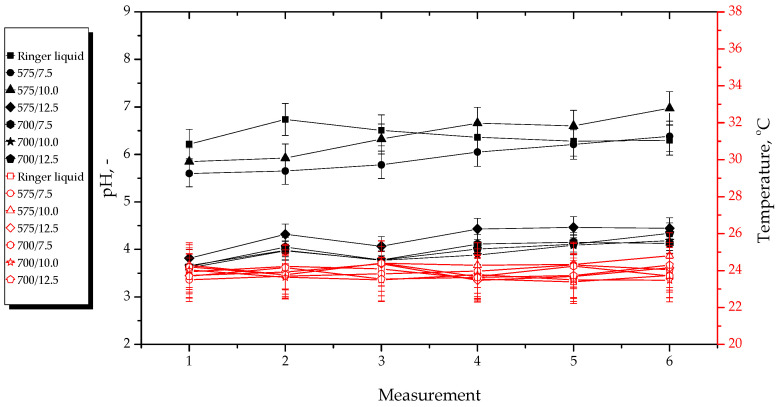
pH and temperature measurements during incubation of hydrogels in Ringer liquid.

**Figure 14:**
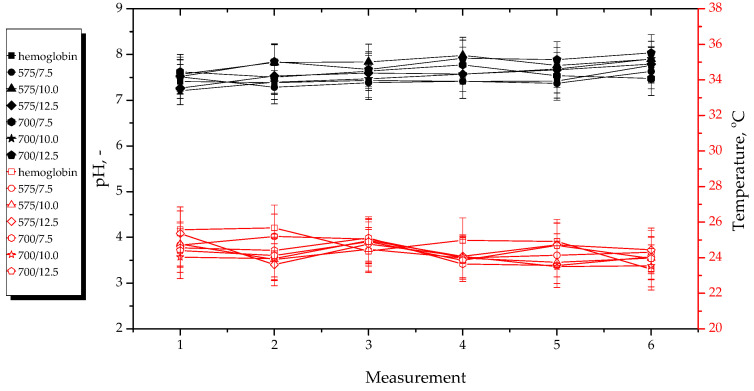
pH and temperature measurements during incubation of hydrogels in hemoglobin.

Results of pH and temperature measurements performed during hydrogels’ incubation in selected simulated physiological liquids are presented below in Figure 11, Figure 12, Figure 13 and Figure 14. The study was conducted in triplicates for each sample, while its results are shown as an average value and an error bar representing the standard deviation (SD).

In the above-presented Figure 11, Figure 12, Figure 13 and Figure 14, the slight differences between the values of both measured parameters for tested samples may be observed. This indicates that the developed materials showed stability in all tested environments. In turn, the lack of rapid changes in pH values of incubation media proves that the hydrogels did not degrade at the time when the study was performed.

The materials incubated in distilled water reduced the pH value of this liquid. The reason of this pH change from approximately seven to approximately three was probably the release of the modifying agents (i.e., *Aloe vera* juice and vitamin C) from the hydrogel matrix because both these additives show acidic pH. The lack of change in pH of SBF was probably due to the high concentration of Ca^2+^ ions in this liquid. They interacted with the functional groups present within the polymer structure, thus increasing its crosslinking density and preventing the release of active substances in such an amount which could change the pH value of this incubation medium. In turn, Ringer liquid contains significantly less divalent ions (such as Ca^2+^), therefore, the release of the modifying substances from the materials consisting of a polymer network with longer chains (thus bigger distances between them) obtained using a crosslinking agent with an average molecular weight of 700 g/mol probably took place. This, in turn, led to the decrease of its pH. Such a phenomenon was not observed in the case of materials obtained using a crosslinker with an average molecular weight of 575 g/mol. This was probably due to the fact that the distances between the polymer chains within this material were too small, and their structure was too densely crosslinked. In the case of 2% hemoglobin solution, any changes in its pH during the incubation of hydrogels in this liquid were not observed. This solution—contrary to the SBF and Ringer liquid—does not contain ions. On the other hand, this liquid contains proteins that probably interacted with functional groups within the polymer structure, therefore, the release of *Aloe vera* juice and vitamin C was not possible.

Importantly, analysis of the results of performed incubation studies allowed to report that tested materials showed buffering properties because during the initial measurements, the pH values of the incubation media increased slightly and then decreased until it stabilized at a constant level. Moreover, a constant, slight increase in pH value of the tested liquids in the case of most hydrogels may be observed. However, this may be due to the absorption of various compounds from the air, e.g., gases.

### 3.3. The Impact of the Hydrogels’ Incubation on Their Chemical Structure Verified Using FT-IR Spectroscopy

**Figure 15:**
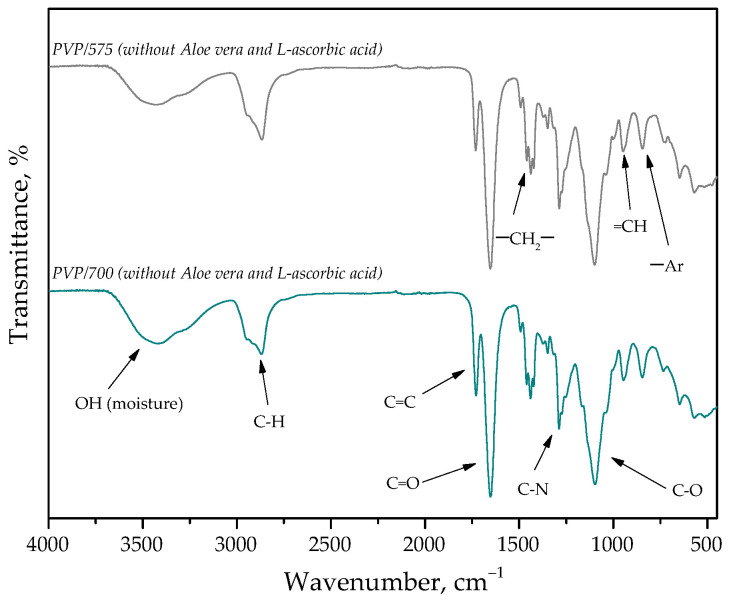
FT-IR spectra of PVP-based hydrogels without any additives.

**Figure 16:**
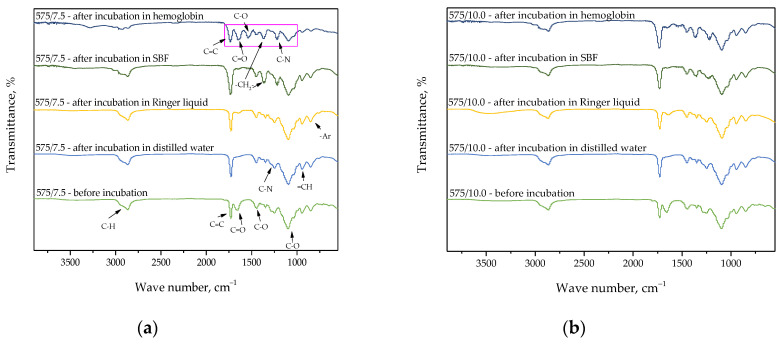

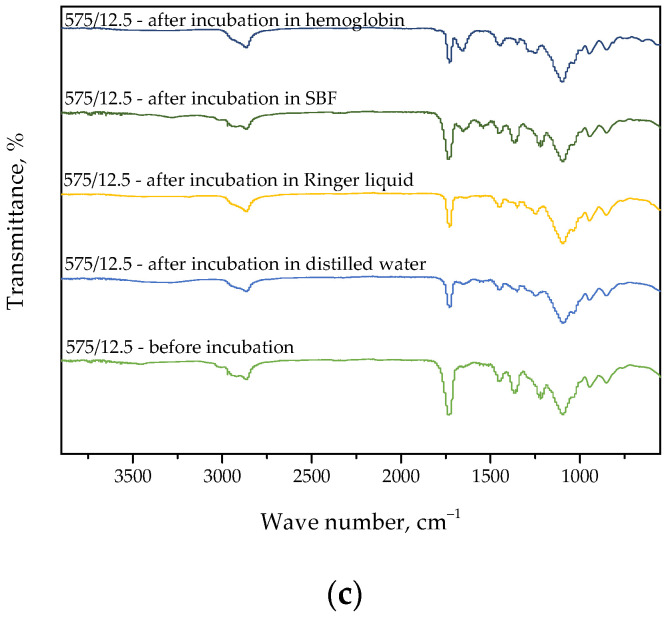
FT-IR spectra showing the impact of the incubation on the structure of sample 575/7.5 (**a**), 575/10.0 (**b**), and 575/12.5 (**c**).

**Figure 17:**
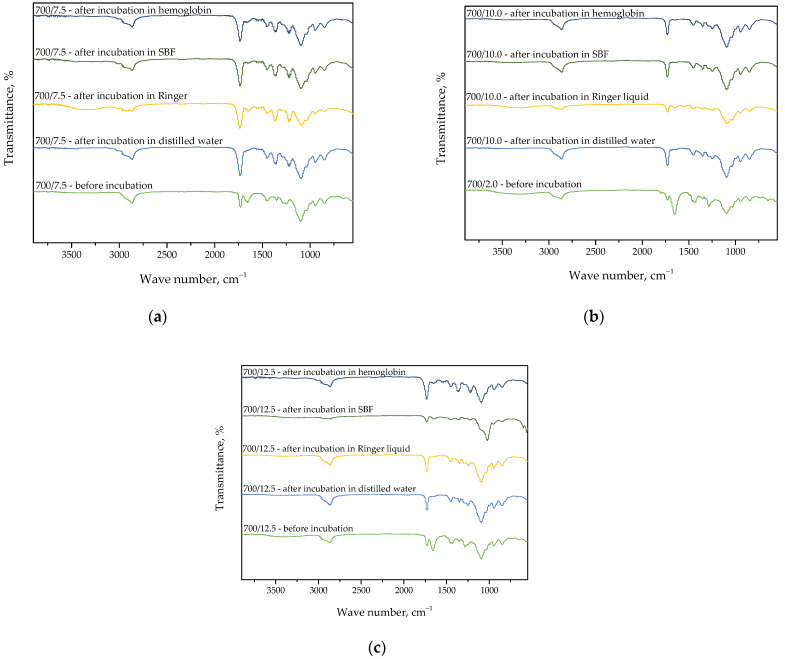
FT-IR spectra showing the impact of the incubation on the structure of sample 700/7.5 (**a**), 700/10.0 (**b**), and 700/12.5 (**c**).

Below, in Figure 15, Figure 16 and Figure 17, FT-IR spectra of samples subjected to the incubation studies are presented. Performed analysis was aimed at verifying the impact of such an incubation on the hydrogels’ structure.

Spectroscopic analyses were performed to characterize the structure of hydrogel materials with various content of the crosslinking agent with an average molecular weight of 575 g/mol and 700 g/mol. The absorption bands observed on FT-IR spectra along with the type of vibration and the chemical structure assigned to them are presented in Table 2.

The vibrations presented in Table 2. and the corresponding chemical bonds derive from the components included in the polymer matrix. Furthermore, FT-IR spectroscopy allowed to confirm the presence of the modifiers introduced into the polymer matrix, which contain the presented bonds in their structure.

Analysis of the results of FT-IR spectroscopy shows a lack of significant differences between the intensities of the absorption bands on obtained FT-IR spectra. This may indicate that the photopolymerization process proceeded properly. On the FT-IR spectra, the bands characteristic for polyvinylpyrrolidone (PVP), diacrylate poly(ethylene glycol) (PEGDA), and 2-hydroxy-2-methylpropiophenone at wave numbers of approximately 1100 cm^−1^, 1750 cm^−1^, and 2850 cm^−1^ may be observed. Slight differences in the intensities of the absorption bands deriving from the hydrogel samples after incubation compared to the same bands of these samples before the study testify to the negligible degradation of tested materials in the incubation media.

Higher intensity of bands at wave numbers of 1100 cm^−1^ and 1750 cm^−1^ observed in the case of samples 575/7.5, 575/10.0, and 575/12.5 as well as 700/7.5, 700/10.0, and 700/12.5 is probably due to the increase in the amount of the crosslinking agent from which these bands derive. On the spectra of the hydrogels before the incubation, the absorption band at approximately 1700 cm^−1^ characteristic for polysaccharides included in *Aloe vera* juice may also be noticed. However, this band may not be observed on the spectra of the materials after the incubation, which is likely due to the release of this modifier from the hydrogel matrix during the study.

### 3.4. Swelling Ability of Hydrogels

**Figure 18:**
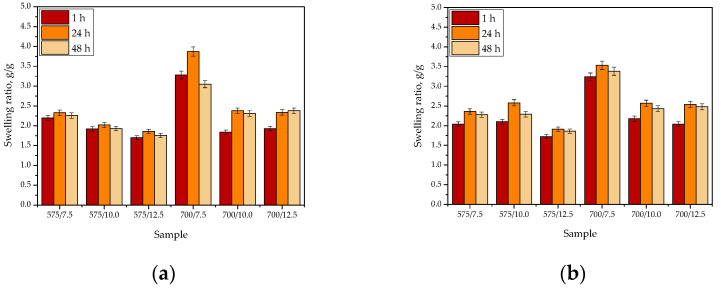

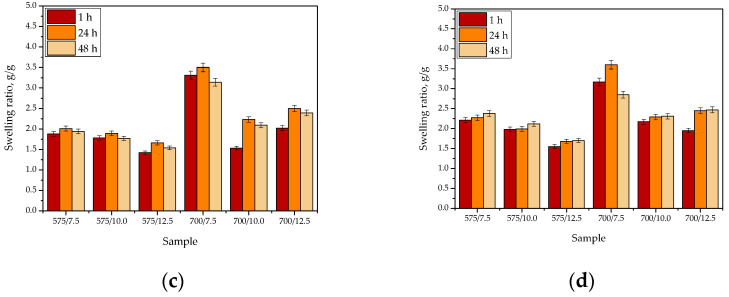
Results of investigations on sorption properties of hydrogels in distilled water (**a**), SBF (**b**), Ringer liquid (**c**), and hemoglobin (**d**).

Results of the research on swelling properties of the hydrogels are shown below in Figure 18. The study was performed in triplicates, and the results are presented as an average value with an error bar representing the standard deviation (SD).

Hydrogels prepared using various amounts of the crosslinking agent with an average molecular weight Mn of 575 g/mol (PEGDA 575) and 700 g/mol (PEGDA 700) were subjected to the sorption investigations, wherein the analyses were tested in distilled water, SBF, Ringer liquid, and 2% hemoglobin solution. The purpose of this study was to verify the impact of both the average molecular weight of the crosslinker applied during the photopolymerization process and the medium in which the swelling takes place on hydrogels’ sorption capacity.

Thus analyzing the results of performed investigations, it may be concluded that as the amount of the crosslinker in the reaction mixture treated with UV radiation increased, the swelling ratio of such obtained hydrogel decreased. This is a result of a higher crosslinking degree of a hydrogel matrix—more specifically the polymer chains forming the polymer matrix—thus the distances between the polymer chains were smaller. Therefore, the penetration of the liquid between the spaces between the chains was significantly limited, which resulted, in turn, in lower swelling ability of such a material and in the lower value of its swelling ratio compared to the value of this parameter obtained for the sample obtained using a lower amount of the crosslinker. However, the described dependance did not hold up in the case of the hydrogel containing 2 mL of the crosslinking agent PEGDA 575 (i.e., sample 575/10.0) analyzed in SBF. Probably, the material with such a composition showed properties promoting SBF sorption. Next, slight differences between swelling properties of samples 700/10.0 and 700/12.5 may be noticed. The swelling ratios calculated for these hydrogels show little difference, wherein their slightly higher value was reported for samples containing the highest content of the crosslinker (i.e., sample 700/12.5). Additionally, analyzing the values of the swelling ratios calculated for samples containing PEGDA 700, a significant jump in the value of the swelling ratio between sample 700/7.5 and samples 700/10.0 and 700/12.5 may be observed. This may be due to the lack of an appropriate polymer chain relaxation within the materials containing higher amount of the crosslinker, which led to the difficulties in the polymer chains loosening and thus to its lower swelling ability. Similar dependance between the polymer chains relaxation and the swelling properties was also reported in [35,36].

Importantly, it may be concluded that the swelling ratio of tested hydrogels depends strongly on the type of crosslinker used during their synthesis. The materials obtained using a crosslinking agent with an average molecular weight 700 g/mol showed higher swelling ability than the materials synthesized using PEGDA with an average molecular weight of 575 g/mol. This is due to the fact that the distances between the polymer chains within the structure of the material obtained using a crosslinker with higher average molecular weight were bigger, thus the solutions may penetrate the structure of the hydrogel material more easily and in larger amounts. The conclusion concerning the impact of the average molecular weight of the crosslinker on hydrogels’ swelling properties is consistent with the conclusions presented in [37].

Moreover, it may also be reported that the liquid in which the study was performed also strongly affected the value of hydrogels’ swelling ratios. Tested materials showed the highest values of the swelling ratio in distilled water, with the lowest ones in SBF and Ringer liquid. Thus, it may be stated that the more ions in the absorbed solution, the lower swelling ratio of the hydrogel tested in this solution. SBF and Ringer liquid—contrary to the distilled water—contain numerous ions (including e.g., Ca^2+^) that may incorporate within the structure of the polymer network. This results in the increase in its crosslinking density and thus in the decrease in its swelling properties. Such an impact of ions on hydrogels’ swelling properties was also described in [38,39,40].

Based on the performed studies, it may also be concluded that the tested materials showed high swelling ability just after 1 h of the study, and a slight increase in the swelling ratio after 24 h of the research compared to the value of this parameter calculated after 1 h. Importantly, the swelling ratio value decreased after 48 h of the research compared to its value determined after 24 h. Therefore, it may be concluded that after 24 h tested materials absorb the maximum amount of the liquid. In turn, the decrease in the samples’ mass in the next part of this study (after 48 h) may indicate the release of the active substance from the hydrogel matrix or the degradation of the analyzed sample in absorbed medium. Nonetheless, the disappearance of the absorption band, which is characteristic for polysaccharides included in the *Aloe vera* juice, reported in the discussion over the results of FT-IR spectroscopy presented in Section 3.3. of this paper, allows to conclude that the release of this modifier probably took place. In the case of samples’ degradation, the disappearance or even a significant decrease in the intensity of more absorption bands (not only these ones deriving from polysaccharides) would be visible.

### 3.5. Mechanical Characteristics of Hydrogels

**Figure 19:**
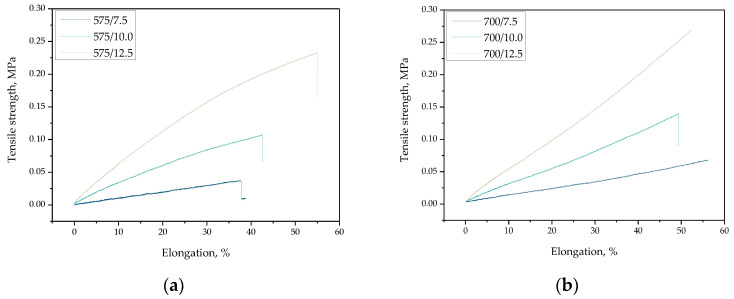
Stress–strain curves of hydrogel samples obtained using various amount of PEGDA 575 (**a**) and PEGDA 700 (**b**).

**Figure 20:**
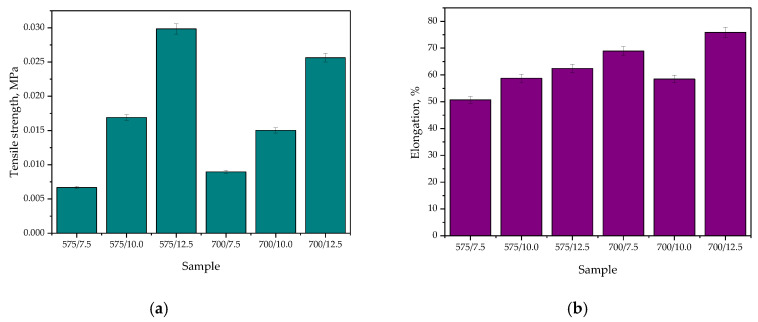
Mechanical characteristics of hydrogels, i.e., the tensile strength (**a**), and percentage elongation (**b**).

The results of performed mechanical studies are shown below, wherein they are presented as the stress–strain curves of all tested hydrogel samples (Figure 19) and as bar charts showing the tensile strength and the percentage elongation of hydrogels (Figure 20).

Based on the performed mechanical experiments, it may be concluded that the highest impact on the tensile strength of analyzed hydrogels was on the amount of crosslinking agent used during their synthesis. The dependance visible in Figure 20a demonstrated that as the amount of the mentioned reagent increased, the tensile strength of hydrogels also increased. The increase in the amount of PEGDA with an average molecular weight of 575 g/mol in the reaction mixture from 7.5 mL to 12.5 mL resulted in approximately five times greater hydrogel’s tensile strength. Such a dependance may also be observed for materials for the synthesis of which a crosslinking agent with an average molecular weight of 700 g/mol was used. The explanation of obtained results may be the fact that the crosslinking agent binds with the polymer chains during the photopolymerization process which, in turn, leads to the formation of crosslinks within the polymer network. Thus, the use of a high amount of this reagent results in higher crosslinking density of the polymer structure and thus in its higher tensile strength. Importantly, the same dependance may be observed in the case of PEGDA 575. Such a conclusion was also reported by Haryanto et al. [41].

Considering the results presented in Figure 20b, it may be reported that both the amount of the crosslinking agent used during the hydrogels’ synthesis and its average molecular weight affected the percentage elongation of hydrogels. For most of the tested materials, as the amount of PEGDA used increased, the percentage elongation of hydrogels also increased. The only exception is sample 700/10.0, which was characterized by the elongation of about 60%, while the samples 700/7.5 and 700/12.5 showed the elongation 70% and 75%, respectively. This may be due to e.g., improperly performed photopolymerization process—i.e., the material might have been subjected to the UV radiation for too long a period, and as a result, such a hydrogel is more fragile than the rest of the tested materials. It should also be reported that the sample 700/7.5 showed higher percentage elongation than sample 575/12.5, while its tensile strength was similar to the value of this parameter determined for sample 575/7.5. The justification for such dependencies may be found in differences in the structure of PEGDA. The crosslinking agent with a higher average molecular weight forms longer crosslinks between the polymer chains, which may result in a higher percentage of such formed polymer network during its stretching.

## 4. Conclusions

▪ Developed hydrogels showed swelling properties in all tested liquids. The highest swelling ratios—i.e., within the range 3.0—4.0 g/g—were achieved by the hydrogel containing the lowest amount of crosslinking agent with an average molecular weight of 700 g/mol in distilled water. This was due to the fact that distilled water, contrary to the other tested liquids, does not contain any ions or proteins that might increase the crosslinking density of the polymer network. Additionally, the crosslinking agents with higher molecular weight form longer crosslinks between the polymer chains, thereby enabling the penetration of a larger amount of liquid.
▪ Hydrogels demonstrated stability both in distilled water and in simulated physiological liquids. No tested sample degraded during the 12 day-incubation, which was confirmed by the lack of significant jumps in the pH values of incubation media. Moreover, hydrogels showed buffering properties in tested liquids.
▪ FT-IR spectroscopy confirmed that the degradation of hydrogels in incubation media did not occur. Importantly, a decrease in the intensity of the absorption band deriving from polysaccharides included in *Aloe vera* juice on the FT-IR spectra of hydrogels after incubation was observed. This was probably due to the release of this active substance from the hydrogel matrices during their incubation.
▪ As the amount of the crosslinking agent in the hydrogels increased, their tensile strength increased. The increase in the amount of the crosslinking agent from 1.5 mL to 2.5 mL resulted in five times higher tensile strength of the hydrogel.
▪ As the amount of the crosslinking agent in the hydrogels decreased, the smoother the hydrogel surface became.
▪ Developed hydrogels showed promising properties in terms of their potential use as innovative dressing materials—i.e., sorption capacity and stability in simulated physiological liquids. Thus, they may be subjected to the detailed biological investigations aimed at further verifying their potential for diabetic foot ulcer treatment.

## Figures and Tables

**Table 1:** Detailed compositions of the hydrogels.

Sample	15% PVP Solution, mL	*Aloe vera* Juice, mL	5% Vitamin C Solution, mL	PEGDA 575, mL	PEGDA 700, mL	Photoinitiator, mL
575/7.5	35	15	10	7.5	-	0.25
575/10.0				10.0		
575/12.5				12.5		
700/7.5				-	7.5	
700/10.0					10.0	
700/12.5					12.5	

**Table 2:** The absorption bands observed on FT-IR spectra of hydrogels before and after the incubation studies.

Wave Number, cm^−1^	Chemical Structure Assigned to the Absorption Band	Type of the Vibration
2850	C-H	stretching
1750	C=O	stretching
1100	C-O	stretching

## Data Availability

Data sharing is not applicable for this article.
